# 

**Published:** 1884-12

**Authors:** 


### CONTENTS:

Art. I.—Materials for Separating Teeth. By D. Genese, D. D. S. .... 337

Art. II—Preparation of the Mouth for the Insertion of Artificial Teeth. By W. H. Atkinson, M. D., D. D. S. .... 340

Art. III—Relative Merits of Watt's Crystal Gold Among Filling Materials By J. F. P. Hudson, D. D. S .... 352

Art. IV.—Causes of Discoloration in Gold Fillings in Teeth. By W. H. Rollins, D. D. S. .... 359

Art. V.—Loose Inferior Incisors and Treatment. By J. Hardman, D. D. S. .... 363

Art. VI.—Micrococci in Relation to Wounds, Abscesses. and Septic Processes. By W. Watson Cheyne .... 369

Art. VII.—Upon Section Plates. By D. Genese. D. D. S .... 371

Art. VIII.—The Influence of Micro-Organisms in Producing Caries of the Teeth. By Arthur Underwood, M. C. R. S., L. D. S. .... 372

New Dental Journals .... 375

### EDITORIAL, ETC.

The New Local Anæsthetic—Hydrochlorate of Cocaine .... 376

Carbolized Potash as an Obtunder .... 377

The Electric Mouth Lamp .... 377

### BIBLIOGRAPHICAL.

Index to Vols. 1 to X X I V of the Dental Cosmos .... 378

The Physicians Visiting List for 1885 .... 378

Permanganate of Potash .... 378

The Dry Treatment of Chronic Suppurative Inflammation of the Middle Ear .... 379

A New Method of Recording the Motions of the Soft Palate .... 379

The Muriate of Cocaine in Opthalmic Surgery .... 379

Dental Caries .... 379

### OBITUARY.

R. Brooke Gwathmey .... 380

### MONTHLY SUMMARY.

Amalgam Notes .... 381

For Sensitive Dentine .... 382

Consolidation .... 382

Tetanus Treated by Ether Spray .... 383

Sulpho Carbol—The New Antiseptic .... 384

